# A coumarin derivative-Cu^2+^ complex-based fluorescent chemosensor for detection of biothiols†

**DOI:** 10.1039/d0ra05651k

**Published:** 2020-10-01

**Authors:** Nguyen Khoa Hien, Mai Van Bay, Phan Diem Tran, Nguyen Tan Khanh, Nguyen Dinh Luyen, Quan V. Vo, Dang Ung Van, Pham Cam Nam, Duong Tuan Quang

**Affiliations:** Mientrung Institute for Scientific Research, Vietnam Academy of Science and Technology Hue 530000 Vietnam; University of Education, Hue University Hue 530000 Vietnam dtquang@hueuni.edu.vn; The University of Danang −University of Science and Education Danang 550000 Vietnam; Faculty of Pharmacy, Hue University of Medicine and Pharmacy Hue 530000 Vietnam; Faculty of Chemical Technology-Environment, The University of Danang−University of Technology and Education 48 Cao Thang Danang 550000 Vietnam; Hoa Binh University Hanoi 100000 Vietnam; The University of Danang −University of Science and Technology Danang 550000 Vietnam pcnam@dut.udn.vn

## Abstract

Herein, a novel fluorescent sensor has been developed for the detection of biothiols based on theoretical calculations of the stability constant of the complex between a Cu^2+^ ion and (*E*)-3-((2-(benzo[*d*]thiazol-2-yl)hydrazono)methyl)-7-(diethylamino) coumarin (BDC) as a fluorescent ligand. In this study, on the basis of density functional theory method, the Gibbs free energy of ligand-exchange reaction and the solvation model were carried out using thermodynamic cycles. The obtained results are in good agreement with the experimental data. The BDC–Cu^2+^ complex can be used as a fluorescent sensor for the detection of biothiols in the presence of non-thiol containing amino acids, with a detection limit for cysteine at 0.3 μM. Moreover, theoretical calculations of excited states were used to elucidate variations in the fluorescence properties. The computed results show that the excited doublet states D_2_ and D_1_ are dark doublet states, which quench the fluorescence of the complex.

## Abstract 2

This study opens a new research direction towards the utilization of complexes between metal ions and fluorescent ligands for the detection of biothiols based on the theoretical calculations of stability constants.

## Graphical abstract

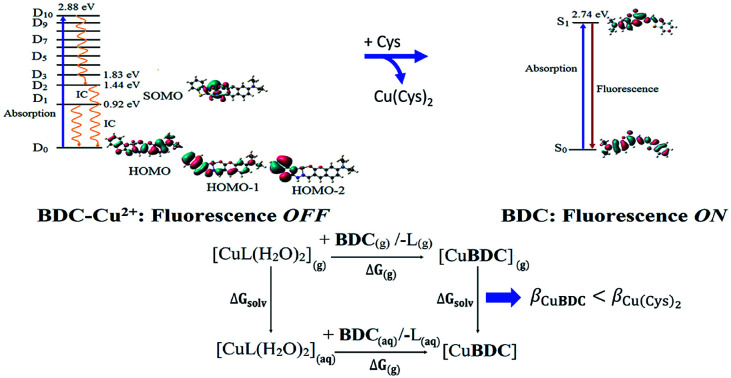

## 1. Introduction

Cysteine (Cys), homocysteine (Hcy), and glutathione (GSH) are thiol biomolecules that play an important role in human biological processes. Some diseases are believed to be related to abnormalities in the concentrations of these compounds in biological systems. A low level of Cys may cause a decline in the immune system, resulting in diseases such as infections, cancer, Parkinson's, Alzheimer's, retarded growth, liver damage, and skin lesions.^1–4^ A high level of Hcy is believed to be associated with heart disease, thromboembolic disease, stroke, atherosclerosis, renal and thyroid dysfunction, psoriasis, diabetes, and cancer.^5,6^ Disturbances in GSH homeostasis are associated with some diseases including immune diseases, inflammatory, cancer, metabolic diseases, diseases of aging, cystic fibrosis, cardiovascular, and neurodegenerative diseases.^1,7^ As a result, the development of selective and sensitive detection methods for biothiols has been attracting the attention of scientists. In particular, analytical methods based on fluorescence technique have been widely developed due to their outstanding advantages.^8,9^ This technique, in particular, can be used to detect biothiols in living cells.^10,11^ A number of fluorescent sensors have been reported based on various interactions with biothiols, such as Michael addition,^12,13^ addition-cyclization with acrylates or aldehydes,^14,15^ cleavage reactions of sulfonamide, sulfonate ester, disulfides,^16–18^ substitution reactions,^19^ and disulfide exchange reactions.^20^ Recently, sensors for biothiols have been widely synthesized and reported; their working mechanism is based on the reactions between biothiols and complexes of fluorescent ligands with metal ions.^21,22^ This approach has opened a new research direction taking advantage of complexes between fluorescent sensors and metal ions towards the detection of biothiols. These works become more convenient if the stability constants of complexes can be determined. Recently, some theoretical computational models for determining the stability constants of complexes have been proposed with the aim of replacing traditional experimental methods.^23–25^ However, the accuracy of these models needs to be verified before their application. This problem can be effectively solved by combining theoretical calculations and empirical investigations to determine the stability constant of complexes.

In the present study, a complex between a Cu^2+^ ion and a fluorescent ligand is reported as a novel chemosensor for the detection of biothiols based on complex exchange reactions. The stability constant of the complex, also known as the complexation equilibrium constant in aqueous solutions, was calculated using the Gibbs free energy of the ligand-exchange reaction determined by a solvation model based on density (SMD), a density functional theory (DFT) method, and thermodynamic cycles.^26,27^ The calculated results are in good agreement with the experimental data obtained by a nonlinear curve-fitting method.^28,29^ From the value of the stability constant, the applicability of the complex as a fluorescent sensor for the detection of biothiols based on the complex exchange reaction was predicted. This was further confirmed by the experimental data obtained by applying this complex as a fluorescent sensor. In addition, herein, theoretical calculations of the excited states were used to explain the fluorescence properties of the substances.

## 2. Materials and methods

### 2.1 Instruments

The Shimadzu RF-5301 spectrofluorophotometer, Shimadzu UV-1800 UV-Vis spectrophotometer, Varian Unity NMR instruments (200 MHz), and electrospray ionization mass spectrometer (ESI MS) were used to obtain the fluorescence spectra, UV-Vis absorption spectra, NMR spectra, and MS spectra, respectively.

### 2.2 Reagents

All chemicals were purchased from Aldrich and used directly without further purification. In particular, 4-(diethylamino)salicylaldehyde, 2-hydrazinobenzothiazole, diethyl malonate, and triethylamine were synthesis grade reagents. Cysteine, glutathione, homocysteine, alanine, aspartic acid, arginine, glycine, glutamic acid, isoleucine, leucine, lysine, methionine, threonine, serine, tyrosine, tryptophan, valine, histidine, HEPES, acetic acid, HCl, NaOH, POCl_3_, and nitrate salts of K^+^, Ca^2+^, Na^+^, Mg^2+^, Zn^2+^, Fe^3+^, Ni^2+^, Pb^2+^, Hg^2+^, Co^2+^, Cd^2+^, and Cu^2+^ were analytical-grade reagents.

All solvents were purchased from Merck. Ethanol was HPLC-grade without fluorescent impurities and used without further purification. DMF was also HPLC-grade; however, it was re-distilled before use. H_2_O was two times distilled water.

### 2.3 Computational methodology

#### 2.3.1 Optimization of the geometries, energies, and absorption and fluorescence properties

Quantum chemical calculations were performed using the Gaussian 09 program package.^30^ The optimization of the geometries of related moieties was carried out using density functional theory (DFT) at the PBE0/6-31+G(d) level of theory.^31–34^ Single-point energies of the optimized geometries in the gas phase were calculated at the higher 6-311++G(d,p) basis set.^35–39^ The variation in the Gibbs free energy (Δ*G*) of reactions was determined by the difference between the sums of the electronic and thermal free energies of products and reactants. The absorption and fluorescence properties were investigated based on the electron excited states of the optimized geometries using the time-dependent density functional theory (TD-DFT) at the PBE0/6-311++G(d,p) level.^40–43^

#### 2.3.2 Theoretical method for determining the stability constants of the complex

**Scheme 1:**
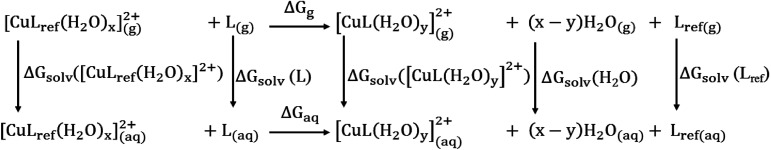
Thermodynamic cycle for the calculation of the Gibbs free energy of a ligand-exchange reaction in an aqueous solution, Δ*G*_aq_.

The stability constant of the complex, also known as the complexation equilibrium constant in aqueous solutions (log *β*), is calculated by the Gibbs free energies of ligand-exchange reactions determined using thermodynamic cycles, the DFT theory method, and the SMD solvent model (Scheme 1).^23–27^ Accordingly, the complexation equilibrium constant in aqueous solutions (log *β*) is calculated by the following equation:1
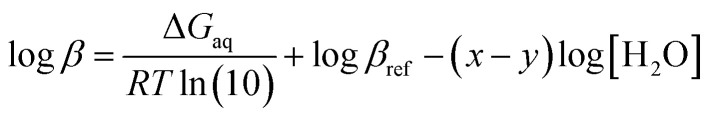
where [H_2_O] is the concentration of water in the standard state, which can be approximated to be 55.56 M.^44,45^ log *β*_ref_ is the experimental equilibrium constant in the aqueous solution of [CuL_ref_(H_2_O)_2_]^2+^, which was used as a reference complex for calculation. Δ*G*_aq_ is the Gibbs free energy of the ligand-exchange reaction in the aqueous solution, which was determined by Scheme 1 and calculated using eqn (2):

2 Δ*G*_aq_ = Δ*G*_g_ + ΔΔ*G*_solv_

Δ*G*_g_ and ΔΔ*G*_solv_ were calculated by eqn (3) and (4), respectively:

3 Δ*G*_g_ = ∑(*Δ*_0_ + *G*_corr_)_products_ − ∑(*Δ*_0_ + *G*_corr_)_reactants_

4 ΔΔ*G*_solv_ = (*x* − *y*)Δ*G*_solv_(H_2_O_2_)_g_ + Δ*G*_solv_([CuL(H_2_O)_*y*_]_g_^2+^) + Δ*G*_solv_(L_ref_)_g_ − Δ*G*_solv_(L)_g_ −Δ*G*_solv_([CuL_ref_(H_2_O)_*x*_]_g_^2+^)

where Δ*G*_g_(the variation in the Gibbs free energy of the reaction) is the difference between the sums of the electronic (*ε*_0_) and thermal free energies (*G*_corr_) of products and reactants in the gas phase at the PBE0/6-311++G(d,p) level of theory and Δ*G*_solv_ is the free energy of solvation of each compound, which was calculated using the solvent model density (SMD) model at the M052x/6-31+G(d) level of theory.^46–49^

#### 2.3.3 Experimental method for determining the stability constants of the complex

The experimental complexation equilibrium constant can also be determined by a nonlinear curve-fitting method.^28,29^ In this case, the experimental complexation equilibrium constant (*β*_ex_) of reaction (5) is calculated by a nonlinear curve-fitting method based on the fluorescence titration spectra of a fluorescent ligand solution (L) with the gradual addition of Cu^2+^ ions.

5 Cu^2+^ + L = [CuL]^2+^

6
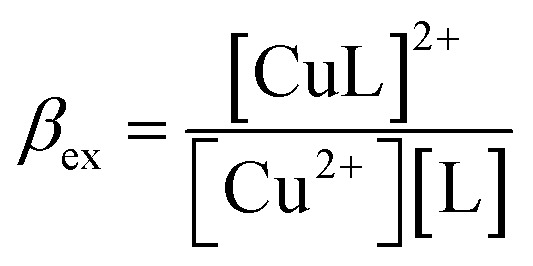


According to this method, the experimental complexation equilibrium constant (*β*_ex_) is detected by the nonlinear curve-fitting method based on the relationship between the two quantities *y* = *C*_M_ and *x* = *F*/*F*_0_, as shown below (see details in the ESI†):7
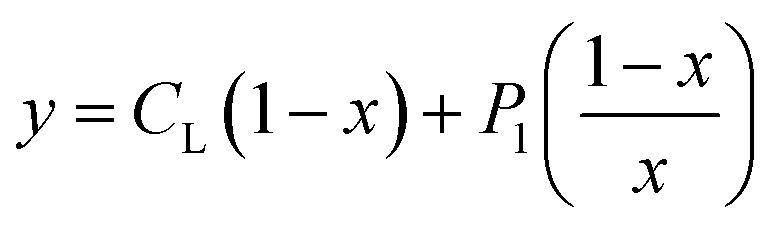
where8
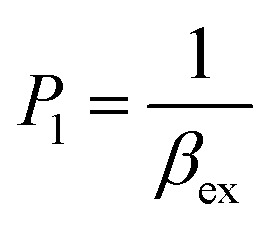


*C*_M_ is the total concentration of the Cu^2+^ ions added to the solution, *F*_0_ is the fluorescence intensity of the free L solution (concentration of L is *C*_L_) at the time when the concentration of Cu^2+^ ions was zero, and *F* is the fluorescence intensity of the L solution at the time when the concentration of Cu^2+^ ions was *C*_M_.

## 3. Results and discussion

### 3.1 Characterization of the fluorescent ligand and its complex with a metal ion

**Fig. 1:**
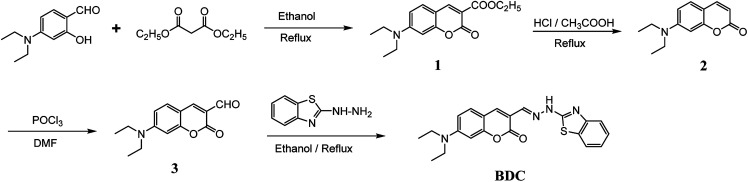
Schematic of the synthesis route of BDC.

(*E*)-3-((2-(Benzo[*d*]thiazol-2-yl)hydrazono)methyl)-7-(diethylamino) coumarin (BDC) was synthesized in about 30% overall yield using 4-(diethylamino)salicylaldehyde *via* the four steps shown in Fig. 1. The structural characteristics of BDC were confirmed by the ^1^H NMR, ^13^C NMR, and mass spectra (ESI†).

**Fig. 2:**
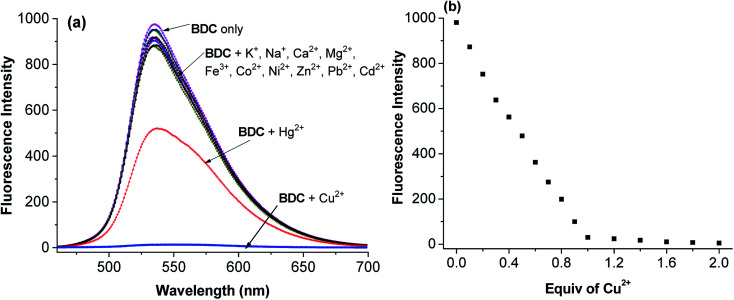
(a) Fluorescence spectra of BDC after the addition of 1 equiv of various metal ions, *i.e.* Na^+^, K^+^, Ca^2+^, Mg^2+^, Fe^3+^, Co^2+^, Ni^2+^, Zn^2+^, Pb^2+^, Cd^2+^, Hg^2+^, and Cu^2+^ ions, and (b) fluorescence titration spectra of BDC with the gradual addition of Cu^2+^ ions (BDC: 5 μM in ethanol/HEPES, pH: 7.4; 1/1, v/v; and excitation wavelength: 460 nm).

The experimental results show that the free ligand BDC is a fluorescent compound. It exhibits a characteristic emission band peaked at 536 nm, with a fluorescence quantum yield (*Φ*) of 0.11 calculated using fluorescein in 0.1 N NaOH (*Φ* = 0.85) as a ref. 50. When 1 equivalent of Cu^2+^ was added to the BDC solution, the fluorescence intensity of the solution was almost completely quenched, over 95%. However, the fluorescence intensity of the BDC solution was quenched only about 40% when 1 equivalent of Hg^2+^ was added. On the other hand, the addition of 1 equivalent of other metal ions, *i.e.* K^+^, Ca^2+^, Na^+^, Mg^2+^, Zn^2+^, Fe^3+^, Ni^2+^, Pb^2+^, Co^2+^, and Cd^2+^ ions, hardly changed the fluorescence spectra of the BDC solution (Fig. 2a). The fluorescence titration spectra of BDC with the gradual addition of Cu^2+^ ions, as shown in Fig. 2b, indicated that the reaction between BDC and Cu^2+^ occurred in a 1 : 1 stoichiometry. In another experiment, when 1 equivalent of EDTA was added to the solution obtained after adding 1 equivalent of Cu^2+^ to the BDC solution, the fluorescence intensity was almost restored to the original fluorescence intensity of the free ligand BDC. These results indicated that the reaction between Cu^2+^ ions and BDC was a reversible reaction and led to the formation of a complex with a 1 : 1 stoichiometry.

**Fig. 3:**
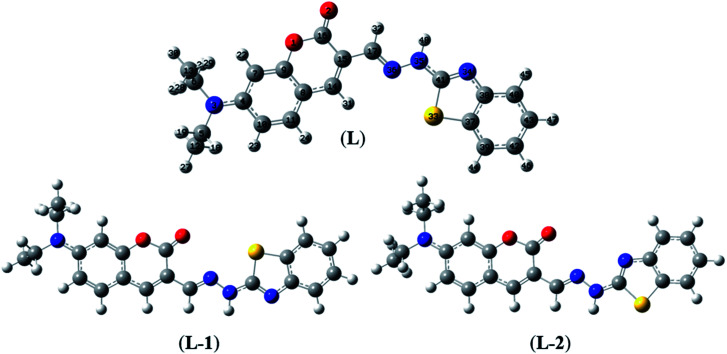
Optimized geometries of BDC at the PBE0/6-31+G(d) level of theory.

**Fig. 4:**
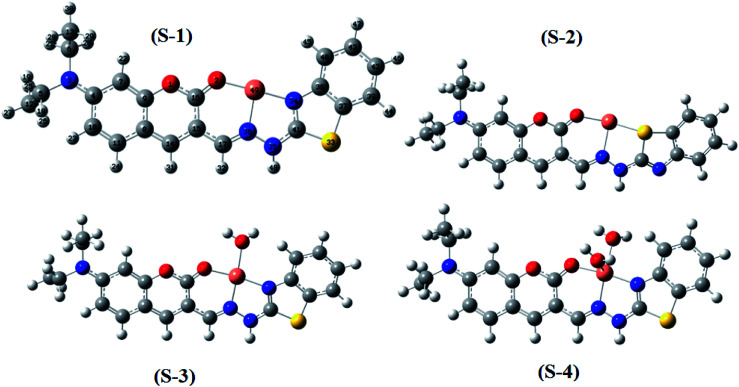
Optimized geometries of the BDC–Cu^2+^ complex at the PBE0/6-31+G(d) level of theory.

The optimization of the geometries of BDC and its 1 : 1 complex with Cu^2+^ was performed using the PBE0 functional with the 6-31+G(d) basis set. The calculated results are presented in Fig. 3 and 4 and Tables S1–S7.†

Herein, three optimized geometries of BDC were defined and denoted as L, L-1, and L-2. In each of these geometries, most atoms are in the same plane except for the atoms of the ethylamino groups. In the L geometry, the configuration of the two chain bonds C16–C15–C17–N36 and N36–N35–C41–N34 is similar to *trans*–*trans* configuration. Moreover, in the L-1 geometry, the configuration of these two chain bonds is similar to *cis*–*trans* configuration. In the L-2 geometry, the configuration of these two chain bonds is similar to *cis*–*cis* configuration. The calculated results show that L is the most energetically favorable geometry. Consequently, the L geometry was used to define the configuration of the complex as well as calculate the Gibbs free energy of the ligand-exchange reaction in the aqueous solution.

Moreover, four optimized geometries of the 1 : 1 complexes between L and Cu^2+^ ion were defined and denoted as S-1, S-2, S-3, and S-4. The molecular formulas of the S-1, S-2, S-3, and S-4 geometries are [Cu(L)]^2+^, [Cu(L)]^2+^, [Cu(L)(H_2_O)]^2+^, and [Cu(L)(H_2_O)_2_]^2+^, respectively. The calculated results show that the reactions for the formation of S-1, S-2, S-3, and S-4 geometries are energetically favorable (Table S8†).

The molecular structures of the S-1, S-2, S-3, and S-4 geometries are presented in Table S9,† including bond lengths, bond angles, and dihedral bond angles, and the coordination numbers of the S-1, S-2, S-3, and S-4 geometries are 3, 3, 4, and 5, respectively. These values are consistent with those reported in previous studies.^41,51–54^ All contact distances of the coordination bonds are significantly shorter than the sum of the van der Waals radii of relevant atoms (Cu: 1.40 Å, N: 1.55 Å, O: 1.52 Å, and S: 1.80 Å). All the four complexes have three coordination bonds between a Cu atom and three atoms of the L ligand. These four atoms are roughly on the same plane, forming the bond angles of approximately 180° and 90°. The formation of complexes mainly leads to changes in the configurations of the two chain bonds C16–C15–C17–N36 and N36–N35–C41–N34 in the BDC moieties. The configurations of the S-2 and S-1, S-3, and S-4 geometries are similar to “*cis*–*trans*” and “*cis*–*cis*” configurations, respectively.

The complexation equilibrium constants in the aqueous solutions of the S-1, S-2, S-3, and S-4 complex configurations were calculated *via* the Gibbs free energies of ligand-exchange reactions determined using the thermodynamic cycles, DFT theory method, and SMD solvent model (Scheme 1). Herein, the reference complex [CuL_ref_(H_2_O)_2_]^2+^ was used for calculation, which is a complex between the Cu^2+^ ions and histamine (L_ref_) as a ligand, and the experimental equilibrium constant in the aqueous solution (log *β*_ref_) of this complex was 9.55 (ref. 55 and 56) (the optimized geometry of histamine (L_ref_) and cartesian coordinate are presented in Fig. S5, S6 and Tables S10 and S11 in the ESI†). The calculation results presented in Table 1 indicate that S-1 is the most stable configuration with a calculated complexation equilibrium constant of 10^7.16^. Consequently, the S-1 configuration was used for all subsequent studies of the BDC–Cu^2+^ complex.

**Table 1:** The calculated complexation equilibrium constants in the aqueous solutions of various complex configurations (energies in kcal)^a^

	Δ*G*_solv_					ΔΔ*G*_solv_	Δ*G*_g_	Δ*G*_aq_	log *β*_ref_	log *β*
	A	B	C	D	E					
S-1	−201.61	−15.35	−172.79	−8.84	−13.50	12.99	−14.47	−1.48	9.55	7.16
S-2	−201.61	−15.35	−152.85	−8.84	−13.50	32.93	−2.08	30.85	9.55	−16.56
S-3	−201.61	−15.35	−157.3	−8.84	−13.50	37.32	−34.03	3.29	9.55	5.40
S-4	−201.61	−15.35	−148.75	−8.84	−13.50	54.71	−42.46	12.25	9.55	0.57

a A: [CuL_ref_(H_2_O)_*x*_]_(g)_^2+^, B: L_(g)_, C: [CuL(H_2_O)_*y*_]_(g)_^2+^, D: H_2_O_(g)_, E: L_ref(g)_.

**Fig. 5:**
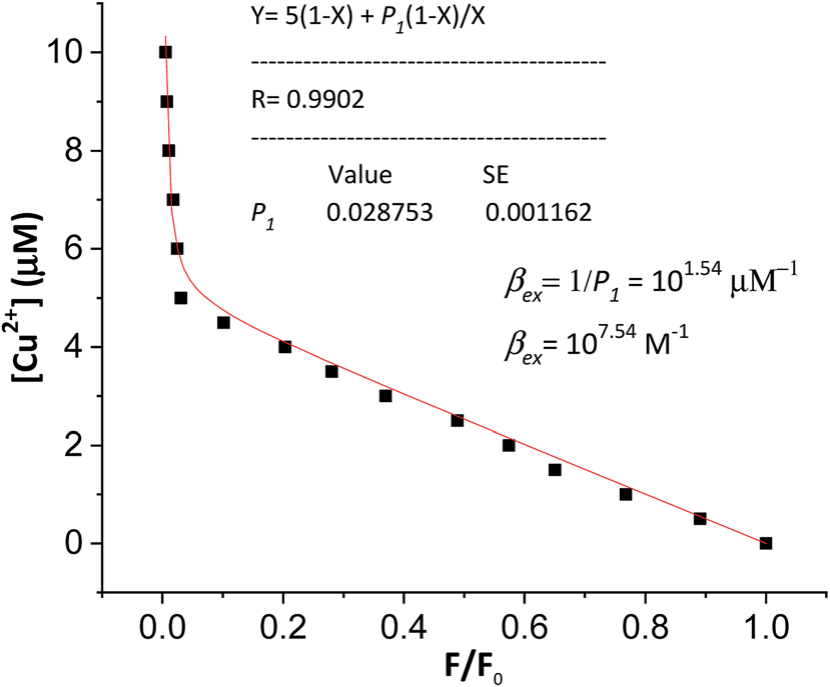
Nonlinear curve-fitting method for the determination of the complexation equilibrium constants in the aqueous solution of the [CuL]^2+^ complex. *C*_M_ is the total concentration of the Cu^2+^ ion added to the solution: 0–10 μM; *F*_0_ is the fluorescence intensity of the free BDC solution (*C*_L_ = 5 μM, in ethanol/HEPES, pH: 7.4, 1/1, v/v); *F* is the fluorescence intensity of the BDC solution at the time when the concentration of Cu^2+^ ions was *C*_M_; excitation wavelength: 460 nm; and emission wavelength: 536 nm.

In order to verify the calculated complexation equilibrium constant before studying the applications of the complex, the experimental complexation equilibrium constant was also determined by a nonlinear curve-fitting method. The fluorescent titration and nonlinear curve-fitting results are shown in Fig. 5. Accordingly, the experimental complexation equilibrium constant was determined to be 10^7.15^ (M^−1^).

This result is in good agreement with the above calculated complexation equilibrium constant. These results provide further important evidence confirming the correctness of the proposed method for the determination of the complexation equilibrium constants in aqueous solutions using DFT theory and the SMD solvent model.

In terms of application, this value of the complexation equilibrium constant of Cu^2+^ ions with BDC is significantly smaller than that of the Cu^2+^ ions with biothiols. For example, the Cu^2+^ ions react with Cys to form a [CuX_2_]^2+^ complex with an equilibrium constant of 10^16.^^57,58^ Moreover, the Cu^2+^ ions react with Hcy to form a [CuHY]^2+^ complex with an equilibrium constant of 10^13.5^.^59^ Compared to the abovementioned reactions, the reactions between the Cu^2+^ ions and GSH are more complicated. In particular, the main reaction occurs between the Cu^2+^ ions and GSH to generate a complex of Cu^+^ ions with GSH in the form of [CuZ_2_]^+^ and introduce the GSSG (oxidized glutathione). Herein, the stability constant of the [CuZ_2_]^+^ complex is substantially large, equal to 10^38.8^.^60–62^ Based on these results, it can be anticipated that the complex of BDC with the Cu^2+^ ions can be used as a fluorescent sensor for the detection of biothiols based on the complex exchange reactions.

### 3.2 Application of the BDC–Cu^2+^ complex in the detection of biothiols

**Fig. 6:**
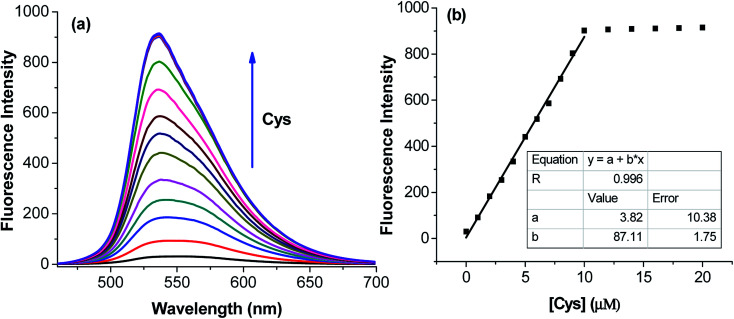
(a) Fluorescence titration spectra of BDC–Cu^2+^ with the gradual addition of Cys and (b) variation in the fluorescence intensity of BDC–Cu^2+^ with the gradual addition of Cys at the emission wavelength of 536 nm. BDC–Cu^2+^: 5 μM, in ethanol/HEPES, pH: 7.4, 1/1, v/v; Cys: 0, 1.0, 2.0, 3.0, 4.0, 5.0, 6.0, 7.0, 8.0, 9.0, 10.0, 13.0, 17.0, and 20.0 μM; and excitation: 460 nm.

**Fig. 7:**
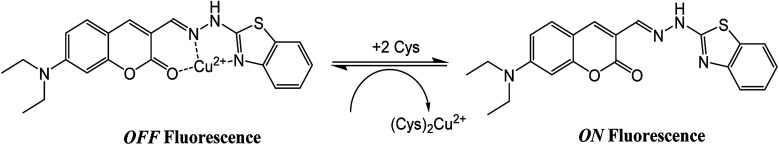
Schematic of the reaction between the BDC–Cu^2+^ complex and Cys.

The application of the BDC–Cu^2+^ complex as a fluorescent sensor for the detection of cysteine was investigated. As shown in Fig. 6, when Cys was gradually added to the solution of the BDC–Cu^2+^ complex, the fluorescence intensity at 536 nm accordingly changed and increased. It almost restored to the original fluorescence intensity of free BDC when Cys was added to the solution at a concentration greater than or equal to twice that of the BDC–Cu^2+^ complex. These results show that the stoichiometry of the reaction between Cys and the BDC–Cu^2+^ complex is 2 : 1, and this reaction leads to the formation of the [CuX_2_]^2+^ complex (X: Cys) and the release of free BDC, resulting in the restoration of fluorescence intensity (Fig. 7). This result is also consistent with those reported in previous studies on the composition of the complex of Cys and Cu^2+^ ion.^57,58^

**Fig. 8:**
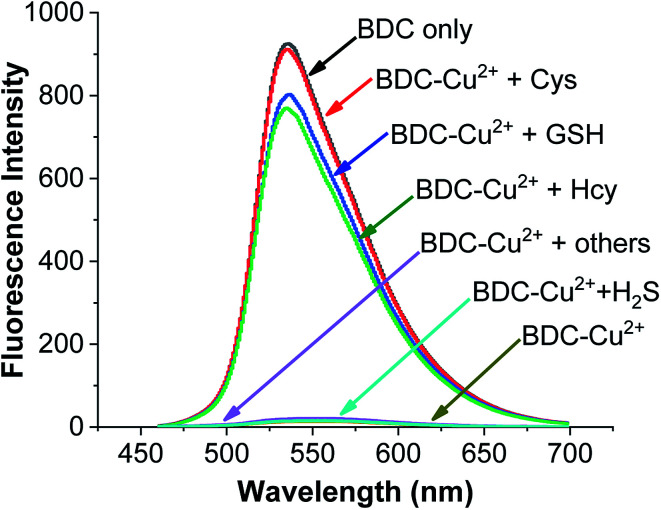
Fluorescence spectra of BDC (5 μM); BDC (5 μM)+Cu^2+^ (5 μM); BDC (5 μM)+ Cu^2+^ (5 μM) + Cys/Hcy/GSH/Ala, Asp, Arg, Gly, Glu, Ile, Leu, Lys, Met, Thr, Ser, Tyr, Trp, Val, His, and H_2_S (10 μM).

To use the BDC–Cu^2+^ complex as a fluorescent sensor for the determination of Cys, the interactions of the BDC–Cu^2+^ complex with H_2_S and other amino acids were also investigated. The experimental results in Fig. 8 show that thiol-containing amino acids, such as GSH and Hcy, also cause fluorescence recovery. Moreover, H_2_S and non-thiol containing amino acids, such as alanine (Ala), arginine (Arg), aspartic acid (Asp), glutamic acid (Glu), glycine (Gly), histidine (His), isoleucine (Ile), leucine (Leu), lysine (Lys), methionine (Met), serine (Ser), threonine (Thr), tyrosine (Tyr), tryptophan (Trp) and valine (Val), did not cause any changes in the fluorescence spectrum of the BDC–Cu^2+^ solution. These results indicate that the BDC–Cu^2+^ complex can be used as a fluorescent sensor for the detection of thiol-containing amino acids in the presence of H_2_S and non-thiol containing amino acids in the concentration range from 0 to 10 μM (from 0 to 2 equivalent). In addition, the experimental results show that the presence of H_2_S at concentrations greater than 10 μM affects the performance of BDC–Cu^2+^ in the detection of biothiols (Fig. S7†).

Similar to the cases of previous studies reported on fluorescent sensors based on complex exchange reactions,^63,64^ the BDC–Cu^2+^ complex also reacted with thiols, including Cys, Hcy, and GSH, and provided similar results. However, in many actual samples, these thiols do not occur simultaneously. For example, in human whole blood, the concentration of reduced glutathione is significantly higher (up to 1 mM) than that of the other thiols. Furthermore, in human plasma, the total concentration of Cys species is up to 250 μM, whereas the concentrations of GSH and Hcy are almost negligible.^65–67^ Therefore, the BDC–Cu^2+^ complex can be used as a fluorescent sensor for the detection of Cys, HCy, or GSH depending on the actual cases.

The applicability of the BDC–Cu^2+^ complex as a fluorescent sensor for the quantification of Cys was also investigated. As shown in Fig. 6b, there is a good linear relationship between the fluorescence intensity of the BDC–Cu^2+^ solution and the concentration of Cys when Cys is added to the BDC–Cu^2+^ solution (5 μM). In the Cys concentration range from 0 to 10 μM, the calibration curve equation was determined as follows: *F*_*I*536_ = (3.8 ± 10.4) + (87.1 ± 1.8) × [Cys], *R* = 0.996. The limit of detection (LOD) and the limit of quantitation (LOQ) of the method were also evaluated by the linear regression method using a calibration curve with a range of low concentrations of Cys (0–5 μM).^68^ The values of these parameters were 0.3 and 1.1 μM, (Fig. S8†). Moreover, the intracellular concentration of Cys was 30–200 μM.^63^ These results indicate that the BDC–Cu^2+^ complex can be used as a fluorescent sensor for the quantification of intracellular Cys and has greater sensitivity than that of the similar previously reported sensors.^69,70^

### 3.3 Theoretical investigation of the changes in the fluorescence properties of the complexes and ligands

**Fig. 9:**
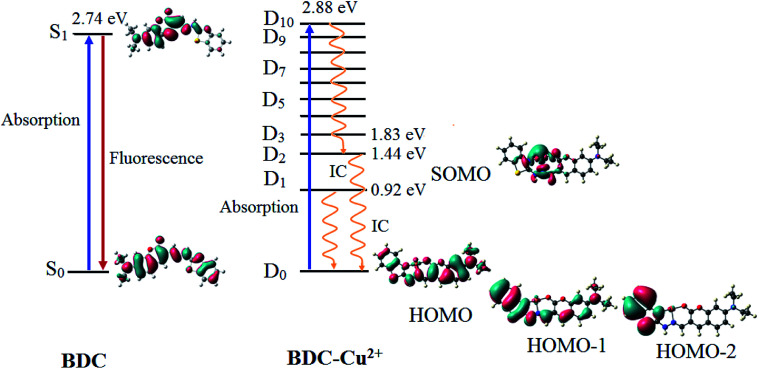
The characteristics of the main transition between the electron excited states and ground states of BDC and BDC–Cu^2+^ at the PBE0/6-31+G(d) level.

The fluorescence properties of the ligand and complex were investigated based on the electronic excited states using time-dependent density-functional theory (TD-DFT) at the PBE0/6-31+G(d) level. The obtained results are presented in Table 2 and Fig. 9.

**Table 2:** The calculated excitation energy (*E*), wavelength (*λ*), and oscillator strength (*f*) for BDC and BDC–Cu^2+^ at the PBE0/6-31+G(d) level of theory

Compound	State	*E* (eV)	*Λ* (nm)	*f*	Contribution of orbital transition	
					Composition	Percentage contribution (%)
BDC	S_0_ → S_1_	2.74	452.9	1.2659	HOMO → LUMO	98.58
	S_0_ → S_2_	3.60	346.4	0.0585	HOMO−1 → LUMO	97.59
	S_0_ → S_3_	3.98	311.6	0.3340	HOMO → LUMO+1	80.64
	S_0_ → S_4_	4.12	301.1	0.1155	HOMO−3 → LUMO	69.20
	S_0_ → S_5_	4.14	299.2	0.0325	HOMO−2→LUMO	90.33
	S_0_ → S_6_	4.40	281.8	0.0293	HOMO−3 → LUMO	15.72
					HOMO → LUMO+2	18.96
					HOMO → LUMO+3	49.74
BDC–Cu^2+^	D_0_ → D_1_	0.92	1350.8	0.0054	HOMO_β_ → SOMO_β_	87.88
	D_0_ → D_2_	1.44	861.9	0.0099	HOMO−2_β_ → SOMO_β_	15.15
					HOMO−1_β_ → SOMO_β_	42.81
	D_0_ → D_3_	1.83	676.7	0.0011	SOMO_α_ → LUMO_α_	30.25
					HOMO_β_ → LUMO_β_	27.33
	D_0_ → D_4_	1.86	666.2	0.0025	HOMO−3_β_ → SOMO_β_	47.21
	D_0_ → D_5_	1.94	637.3	0.0124	HOMO−2_β_ → SOMO_β_	21.27
					HOMO−1_β_ → SOMO_β_	34.64
	D_0_ → D_6_	2.14	579.3	0.0035	HOMO−21_β_ → SOMO_β_	32.03
	D_0_ → D_7_	2.16	574.2	0.0019	HOMO−28_β_ → SOMO_β_	15.67
					HOMO−21_β_ → SOMO_β_	26.27
	D_0_ → D_8_	2.27	545.6	0.0021	HOMO−2_β_ → SOMO_β_	40.68
	D_0_ → D_9_	2.68	462.4	0.0014	SOMO_α_ → LUMO+1_α_	24.45
					HOMO_β_ → LUMO+1_β_	23.65
	D_0_ → D_10_	2.88	430.7	1.2645	SOMO_α_ → LUMO_α_	39.49
					HOMO_β_ → LUMO_β_	41.68
	D_0_ → D_11_	2.89	428.7	0.1655	HOMO−3_β_ → SOMO_β_	63.47
	D_0_ → D_12_	2.97	417.9	0.0949	HOMO−4_β_ → SOMO_β_	66.61
	D_0_ → D_13_	3.13	396.5	0.0001	HOMO_α_ → LUMO_α_	22.56
					SOMO_α_ → LUMO+1_α_	17.29
					HOMO−1_β_ → LUMO_β_	21.26
					HOMO_β_ → LUMO+1_β_	16.81

The results provided in Table 2 indicate that in BDC, the S_0_ → S_1_ transition plays a key role in all the singlet electronic transitions from ground state (S_0_) to excited states (S_*n*_). This is due to the fact that the oscillator strength (*f*) of this transition is 1.2659, substantially larger than those of the other transitions. The S_0_ → S_1_ transition energy is 2.74 eV (452.9 nm). The calculated result is completely consistent with the empirical investigations, indicating that BDC exhibits a maximum absorption peak at 460 nm. The HOMO → LUMO transition plays a key role in all the orbital transitions from S_0_ to S_1_, with a percentage contribution of 98.58%. This transition occurs between two adjacent MOs. Therefore, it can be firmly affirmed that photoinduced electron transfer (PET) does not occur in BDC, and the abovementioned transition leads to a green emission at 536 nm, as observed *via* empirical investigations.^71,72^

The formation of the BDC–Cu^2+^ complex leads to a significant decrease in the energy gap between the ground state and the excited states of BDC. As a result, the energy gap between the excited state and the D_0_ state reaches 2.88 eV (430.7 nm) only when the D_10_ excited state is attained. The D_0_ → D_10_ transition has an oscillator strength of 1.2645, significantly bigger than that of the other transitions; therefore, this transition plays a key role in all the electronic transitions. The D_0_ → D_10_ transition is mainly contributed by the SOMO → LUMO and HOMO → LUMO transitions, with the percentage contributions of 39.49% and 41.68%, respectively. These transitions are believed to lead to a strong absorption spectrum, as observed in the experiments. Regarding the fluorescence properties of the BDC–Cu^2+^ complex, on the basis of Kasha's rule, the fluorescence occurs from the lowest-lying electron excited state D_1_,^73,74^ in which the D_1_ → D_0_ transition is mainly contributed by the LUMO → SOMO. However, the lack of overlapping between the LUMO and SOMO makes the radiation transition D_1_ → D_0_ strongly forbidden. Instead, the internal conversion process occurs from D_1_ to D_0_ as a radiationless transition. Considering the exceptions of the Kasha rule, from the D_2_ state to the D_10_ state, the energy gap between adjacent excited states is quite small, less than 0.42. Thus, according to the energy gap law for radiationless transitions, the internal conversion processes from the excited states at higher energy level to the excited state at adjacent low energy levels quickly occur, competing with the fluorescent radiation process, which quenches the D_*n*_ → D_0_ fluorescence (*n* > 2).^75,76^ Only the energy gap between the D_2_ and D_1_ states is quite large, 0.52 eV, which should be considered. The D_2_ → D_0_ transition is mainly contributed by the SOMO → HOMO−2 and SOMO → HOMO−1 transitions. However, the lack of overlapping between the SOMO and HOMO−2 or HOMO−1 makes the radiation transition D_2_ → D_0_ strongly forbidden. As a result, the IC process is also preferred over the fluorescent radiation process, which quenches the D_2_ → D_0_ fluorescence. In summary, in the BDC–Cu^2+^ complex, since the energy gap between adjacent excited states is small, the transitions from the excited states D_*n*_ (*n* > 2) to the excited state D_2_ are dominated by the internal conversion processes. Moreover, due to the lack of overlapping between MOs during each transition, the D_1_ → D_0_ and D_2_ → D_0_ transitions are dominated by the internal conversion processes. The D_2_ and D_1_ states are dark doublet states that quench the fluorescence.^77^

## 4. Conclusions

In summary, herein, a complex exchange reaction-based fluorescent chemosensor was investigated for the detection of biothiols. This sensor was designed based on the theoretical calculations of the stability constants of the complex between the Cu^2+^ ions and fluorescent ligands. The obtained results are completely consistent with the experimental results. The BDC–Cu^2+^ complex can be used for the detection of biothiols in the presence of non-thiol containing amino acids. The limit of detection and the limit of quantitation of the proposed chemosensor for Cys are 0.3 and 1.1 μM, respectively. This result opens a new research direction toward the utilization of the complexes between metal ions and fluorescent ligands for the detection of biothiols based on the theoretical calculations of the stability constants.

In this study, the theoretical calculations of the excited states were also used to elucidate the changes in the fluorescence properties of compounds. The quenching of the fluorescence of the BDC–Cu^2+^ complex occurred because the internal conversion processes dominated the fluorescence process due to the small energy gap between adjacent excited states, from the excited states D_*n*_ (*n* > 2) to the excited state D_2_. Moreover, the D_2_ and D_1_ states are dark doublet states because of the lack of overlapping between MOs during each transition.

## Conflicts of interest

There are no conflicts to declare.

## Supplementary Material

RA-010-D0RA05651K-s001
