# Supplementary material for: A coumarin derivative-Cu2+ complex-based fluorescent chemosensor for detection of biothiols
Source: RSC Adv. 2020 Oct 1;10(60):36265–74. doi: 10.1039/d0ra05651k (PMC9057049; doi:10.1039/d0ra05651k)
Supplement: RA-010-D0RA05651K-s001 [file RA-010-D0RA05651K-s001.pdf]

Electronic Supplementary Material (ESI) for RSC Advances.
This journal is © The Royal Society of Chemistry 2020

Supplementary Data for

A Coumarin derivative-Cu^{2+} complex-based fluorescent chemosensor for detection of biothiols

Nguyen Khoa Hien[1§], Mai Van Bay[2,3§], Phan Diem Tran[1], Nguyen Tan Khanh[4], Nguyen Dinh Luyen[2], Quan V. Vo[5], Dang Ung Van[6], Pham Cam Nam[7*], and Duong Tuan Quang[2*]

[1] Mientrung Institute for Scientific Research, Vietnam Academy of Science and Technology, Hue 530000, Vietnam

[22] University of Education, Hue University, Hue 530000, Vietnam

[3] The University of Danang–University of Science and Education, Danang 550000, Vietnam

[4] Faculty of Pharmacy, Hue University of Medicine and Pharmacy, Hue 530000, Vietnam

[5] Faculty of Chemical Technology-Environment, The University of Danang–University of Technology and Education, 48 Cao Thang, Danang 550000, Vietnam.

[6] Hoa Binh University, Hanoi 100000, Vietnam

[7] The University of Danang–University of Science and Technology, Danang 550000, Vietnam

*Corresponding authors: dtquang@hueuni.edu.vn; pcnam@dut.udn.vn

[§] These authors contributed equally to the work.

1. Synthesis of (E)-3-((2-(benzo[d]thiazol-2-yl)hydrazono)methyl)-7-(diethylamino) coumarin (BDC)

*** Synthesis of 7-Diethylamino-3-formylcoumarin (3)**

3.86 g of 4-Diethylaminosalicylaldehyde (20 mmol) and 6.40 g of diethylmalonate (40 mmol) were dissolved with 100 mL of absolute ethanol in a 500 ml flask. The resulting solution was added dropwise 5mL of triethylamine. The reaction mixture was stirred and refluxed at room temperature for 6 hours. Then, the solvent was evaporated by rotary evaporators to obtain the crude product of (**2**).

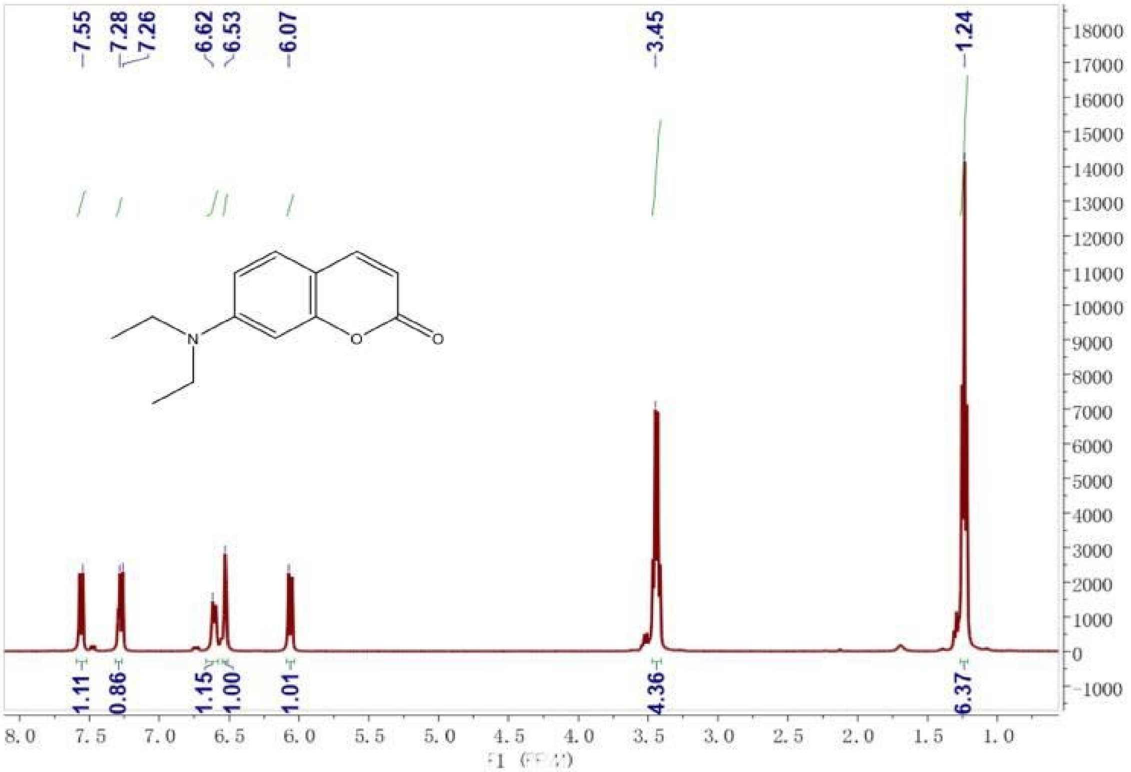

Fig.S1. ^{1}H-NMR data for the product **3**.

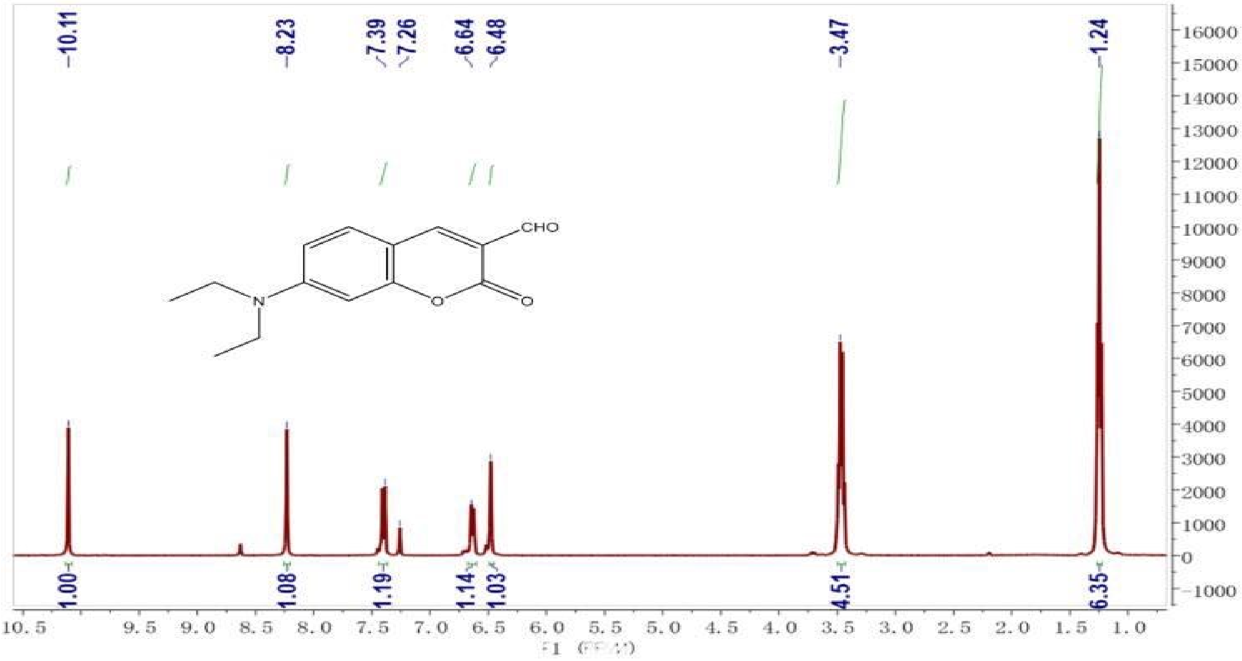

Fig.S2. ^{1}H-NMR data for the product **4**.

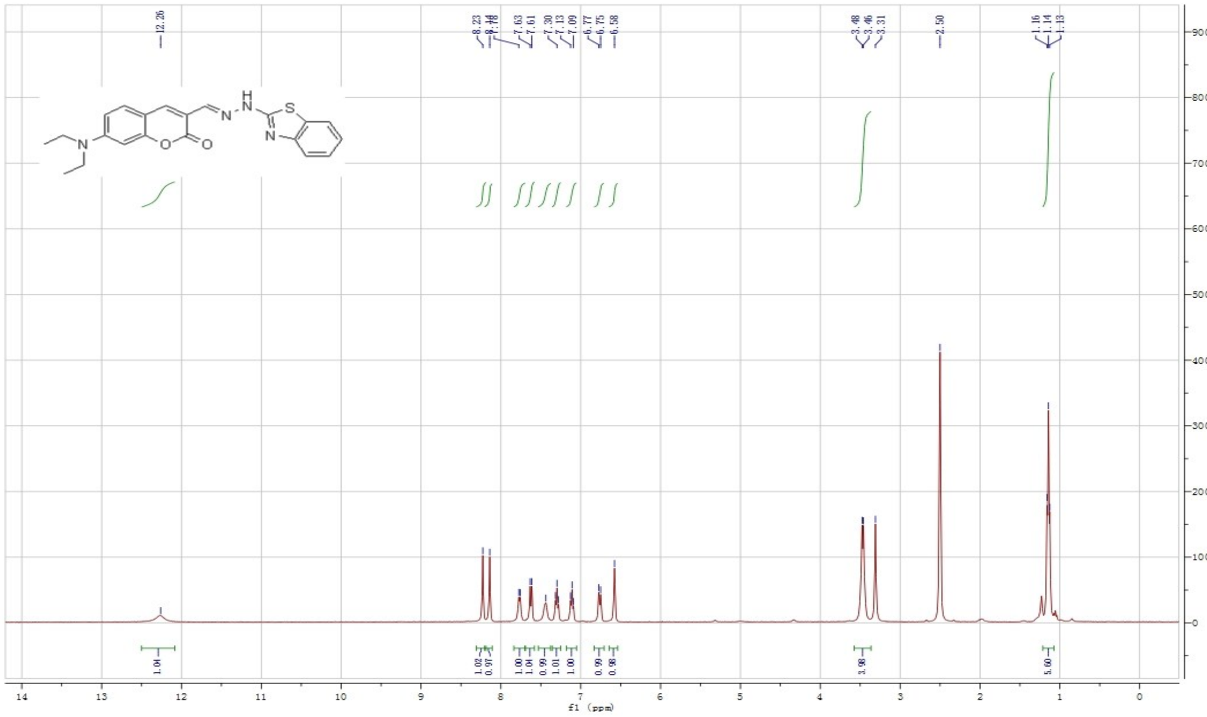

Fig.S3. ^{1}H-NMR data for the probe **BDC**.

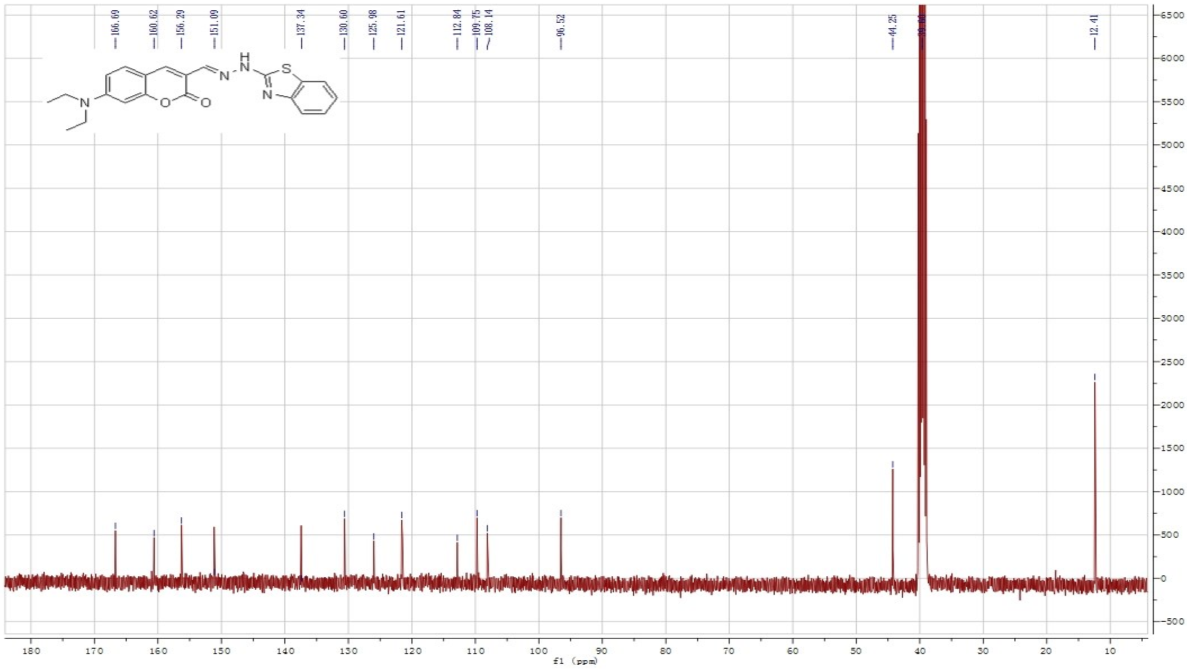

Fig.S4. ^{13}C-NMR data for the probe **BDC**.

2. The Cartesian coordinates for the structures

Table.S1. XYZ coordinates for calculated optimized geometry of structure of **L** at the PBE0/6-31+G(d) level of theory.

O	-2.65981500	2.19220200	-0.15152200
O	-1.10673200	3.76696500	-0.24840900
N	-6.25037400	-0.91934400	0.05670100
C	-4.90690100	-0.66154600	0.04153400
C	-6.77731700	-2.25583500	0.27522000
C	-7.23150800	0.13217000	-0.15310200
C	-4.41739700	0.65834500	-0.04964100
C	-2.10286300	-0.13855000	0.00666700
C	-3.05444500	0.89540500	-0.06801900
C	-3.95128400	-1.71632500	0.11384400
C	-2.60064900	-1.45248700	0.09834000
C	-6.92682000	-3.07292000	-1.00449600
C	-7.63564700	0.85877900	1.12626700
C	-0.73013200	0.21410500	-0.01600600
C	-0.33429800	1.52093800	-0.10276200
C	-1.33652200	2.58114800	-0.17295600
C	1.05083200	1.95429500	-0.13233300
H	-6.14825400	-2.78099000	1.00200700
H	-7.75516500	-2.14360100	0.75854500

H	-6.84999500	0.83875200	-0.89842700
H	-8.11354100	-0.33283300	-0.60930500
H	-5.07480100	1.51828200	-0.08490400
H	-4.27740100	-2.74849500	0.16103500
H	-1.89374900	-2.27757500	0.14879300
H	-7.59806500	-2.57285900	-1.71169900
H	-5.96238400	-3.21331100	-1.50332600
H	-7.34618300	-4.06086000	-0.78158400
H	-8.07063600	0.16173500	1.85139100
H	-6.77550200	1.34154900	1.60109800
H	-8.38348500	1.62965600	0.90766300
H	0.02773100	-0.56525600	0.03679800
H	1.22308900	3.03275900	-0.20759800
S	4.08223900	-0.98838300	0.08081200
N	5.56128500	1.17372600	-0.08145200
N	3.26883300	1.58671800	-0.10820700
N	2.01699500	1.10828400	-0.07340900
C	5.82421700	-1.16822200	0.09123900
C	6.43072600	0.10466100	-0.00374700
C	6.57815200	-2.33466300	0.17565500
C	7.82616400	0.19634500	-0.01300400
C	4.33832400	0.74739900	-0.04814500

C	7.96616500	-2.22240600	0.16489600
C	8.58103400	-0.96707800	0.07135500
H	6.10094100	-3.30827400	0.24818000
H	8.29464700	1.17359800	-0.08612300
H	8.57537100	-3.11991500	0.22989800
H	9.66600900	-0.90218700	0.06461600
H	3.46176400	2.58359300	-0.18086500

Table.S2. XYZ coordinates for calculated optimized geometry of structure of **L-1** at the PBE0/6-31+G(d) level of theory.

O	1.89700100	0.98027300	0.11112500
O	-0.28208900	1.41157700	0.15406700
N	6.64602900	0.46549500	0.03651000
C	5.38506700	-0.08568200	-0.01898900
C	7.85214200	-0.32782600	-0.20069500
C	6.85318700	1.88688800	0.31702600
C	4.22730900	0.71963600	0.07447800
C	2.77481400	-1.24519300	-0.13553500
C	2.96657800	0.14311600	0.01938100
C	5.19856400	-1.49374600	-0.17047600
C	3.93473800	-2.04241600	-0.22810400
C	8.42022500	-0.98501000	1.06319900
C	6.81841900	2.77748900	-0.93134400

C	1.43899500	-1.72714600	-0.18493200
C	0.34980500	-0.89587000	-0.09278900
C	0.55850600	0.55438500	0.06294700
C	-0.98482300	-1.46520000	-0.14976400
H	7.64567100	-1.08072700	-0.96777500
H	8.60170000	0.34251600	-0.63632000
H	6.11145400	2.22018600	1.04961700
H	7.82633000	1.98201200	0.81184300
H	4.27835500	1.79589200	0.16893400
H	6.05358700	-2.15551700	-0.22202400
H	3.82478400	-3.11847700	-0.33918700
H	8.67402300	-0.23055100	1.81606200
H	7.69730100	-1.67412600	1.51110900
H	9.33116800	-1.54679700	0.82591900
H	7.59408900	2.48051600	-1.64606300
H	5.85116400	2.71243200	-1.43896600
H	6.99311200	3.82423100	-0.65725500
H	1.27961700	-2.79841900	-0.30113600
H	-1.01783500	-2.55896700	-0.26141400
S	-4.47461200	0.95113700	0.09689300
N	-5.57222500	-1.44364500	-0.12881300
N	-3.23667000	-1.46743200	-0.14539700

N	-2.07729800	-0.78509900	-0.07859300
C	-6.23597000	0.82352300	0.09459300
C	-6.61670500	-0.53531500	-0.03437900
C	-7.18300700	1.84045900	0.19913800
C	-7.97812800	-0.86587100	-0.05699300
C	-4.43802800	-0.81506400	-0.07450300
C	-8.53378500	1.49098300	0.17431800
C	-8.92468100	0.14946100	0.04755700
H	-6.88096900	2.87911800	0.29773200
H	-8.27003500	-1.90690500	-0.15554600
H	-9.28812000	2.26870500	0.25436300
H	-9.98200500	-0.10078600	0.03059300
H	-3.26707900	-2.48217900	-0.24305900

Table.S3. XYZ coordinates for calculated optimized geometry of structure of **L-2** at the PBE0/6-31+G(d) level of theory.

O	1.99102300	1.08611400	0.09909300
O	-0.17656500	1.57831000	0.13895100
N	6.72333400	0.43583300	0.02951400
C	5.44699500	-0.08047300	-0.01570300
C	7.90649500	-0.39650200	-0.18812100
C	6.97034800	1.85569200	0.28447500
C	4.31285700	0.75890700	0.06499400

C	2.80490500	-1.16667100	-0.11161600
C	3.03571800	0.21811500	0.02032800
C	5.22048600	-1.48493900	-0.14359100
C	3.94126900	-1.99803800	-0.19158700
C	8.45120200	-1.04521500	1.09036200
C	6.95788500	2.72442200	-0.97953500
C	1.45564800	-1.61114200	-0.15290000
C	0.39012300	-0.74909700	-0.07298700
C	0.63827200	0.69745600	0.06012900
C	-0.95867900	-1.28687100	-0.12281700
H	7.68067400	-1.15788200	-0.94123900
H	8.67672200	0.24298700	-0.63401300
H	6.23960200	2.22275800	1.01200300
H	7.94671700	1.93260000	0.77611800
H	4.39472600	1.83460800	0.14202300
H	6.05610000	-2.17175200	-0.18493700
H	3.80050200	-3.07223000	-0.28502200
H	8.72410600	-0.28381700	1.82944700
H	7.70681000	-1.70403100	1.54855600
H	9.34623200	-1.63776800	0.86772600
H	7.72310400	2.39249800	-1.69012900
H	5.98797900	2.67775400	-1.48408400

H	7.16276200	3.77056200	-0.72468400
H	1.26676100	-2.67932900	-0.25273700
H	-1.00732900	-2.38310000	-0.22587200
S	-5.86427500	-1.75452400	-0.16389600
N	-4.64785200	0.57983700	0.06920300
N	-3.19546800	-1.29327200	-0.12224600
N	-2.04030800	-0.59018800	-0.05613700
C	-6.85616800	-0.30396000	-0.01472400
C	-6.01101300	0.82873500	0.09723900
C	-8.24427300	-0.19191200	0.00112900
C	-6.59066000	2.09750900	0.22744700
C	-4.42258300	-0.68331700	-0.05824400
C	-8.79938700	1.08199600	0.13198700
C	-7.97787600	2.21333700	0.24384100
H	-8.88027400	-1.06785300	-0.08543800
H	-5.94451500	2.96525100	0.31334900
H	-9.87985900	1.19377900	0.14682800
H	-8.43099300	3.19569800	0.34499700
H	-3.16442400	-2.30739100	-0.21788600

Table.S4. XYZ coordinates for calculated optimized geometry of structure of **S-1** at the PBE0/6-31+G(d) level of theory.

O	1.89701600	0.66389100	0.10090700

O	-0.24866700	0.91673400	0.19205700
N	6.63621200	0.66313000	0.00112600
C	5.48200400	-0.00102900	-0.04187000
C	7.93949400	0.00772600	-0.18698900
C	6.70324400	2.11082000	0.24130200
C	4.22729000	0.68341700	0.05166600
C	3.02381000	-1.45550300	-0.12101300
C	3.07140700	-0.03630700	0.01143300
C	5.45338200	-1.44003300	-0.18303100
C	4.28189200	-2.12702100	-0.21854700
C	8.52613000	-0.52019600	1.11577400
C	6.62103500	2.92555800	-1.04345800
C	1.79147400	-2.07139400	-0.15048100
C	0.59055900	-1.33067200	-0.05768900
C	0.69605600	0.09203600	0.08083500
C	-0.66347700	-2.00417200	-0.11330500
H	7.84298000	-0.77927500	-0.93881600
H	8.60018200	0.76248600	-0.62328900
H	5.92274000	2.39274200	0.95269100
H	7.65531400	2.29495600	0.74780500
H	4.16470900	1.76143400	0.12804200
H	6.38201100	-1.99353700	-0.23821300

H	4.29188300	-3.20945200	-0.31390800
H	8.65457300	0.28178200	1.84965800
H	7.89404800	-1.29332200	1.56426800
H	9.51132100	-0.95529700	0.92145600
H	7.42958700	2.66435100	-1.73337800
H	5.66946300	2.77404900	-1.56329100
H	6.71571400	3.98985300	-0.80683200
H	1.73724900	-3.15343300	-0.25464900
H	-0.64867100	-3.09638700	-0.15617600
S	-5.72023000	-1.72087100	-0.12757400
N	-3.89883700	0.11089600	0.08620700
N	-3.00233100	-2.02514000	-0.10321600
N	-1.80731000	-1.38319000	-0.13777900
C	-6.22506900	-0.04145800	-0.02746500
C	-5.10611200	0.80509100	0.06571100
C	-7.52280400	0.45924200	-0.01610900
C	-5.27264200	2.18634500	0.17013900
C	-4.08581100	-1.19595500	-0.02245200
C	-7.67591400	1.83830300	0.07466300
C	-6.56518300	2.69013100	0.16646400
H	-8.38600400	-0.19613200	-0.07933700
H	-4.41552800	2.84951300	0.25495300

H	-8.67625100	2.26034400	0.07880500
H	-6.71991800	3.76199100	0.24246800
H	-3.05549400	-2.99188300	0.20501000
Cu	-2.05075000	0.54326500	0.06077100

Table.S5. XYZ coordinates for calculated optimized geometry of structure of **S-2** at the PBE0/6-31+G(d) level of theory.

O	2.05143100	0.77092000	0.14593600
O	-0.08128800	1.14534300	0.19762500
N	6.77890400	0.47551500	0.09936900
C	5.58279600	-0.11628500	0.00848000
C	8.04230500	-0.24569900	-0.11544900
C	6.93305500	1.90157100	0.41862900
C	4.37597400	0.64183200	0.13335000
C	3.04617400	-1.39330800	-0.19099600
C	3.17163600	0.00414900	0.03078300
C	5.47114400	-1.53775400	-0.21622900
C	4.25456100	-2.14180800	-0.30742700
C	8.58520800	-0.86349000	1.16819700
C	6.90964400	2.78200100	-0.82637500
C	1.76772300	-1.93221500	-0.28231400
C	0.61867200	-1.13870900	-0.17439300
C	0.79666000	0.29497600	0.05890700

C	-0.65530100	-1.76927200	-0.27392600
H	7.90365100	-0.99191900	-0.90064400
H	8.74815300	0.49003900	-0.51168600
H	6.16545600	2.19343800	1.13909200
H	7.89272800	1.99781300	0.93472100
H	4.38018900	1.71424400	0.28219700
H	6.36276600	-2.14697400	-0.29161000
H	4.19454400	-3.21488900	-0.46730800
H	8.75420600	-0.10439700	1.93832100
H	7.90636400	-1.61927200	1.57540200
H	9.54534100	-1.34389600	0.95664700
H	7.70667100	2.50903400	-1.52477900
H	5.95365800	2.71425500	-1.35493600
H	7.06617000	3.82424000	-0.53210700
H	1.65580600	-3.00203200	-0.44880400
H	-0.65372100	-2.85759400	-0.38637600
S	-4.11267100	0.62620700	-0.60701700
N	-5.24369000	-1.63183900	0.11402300
N	-2.93968100	-1.79553900	-0.26198300
N	-1.79153000	-1.12477000	-0.23179700
C	-5.79861300	0.66549400	-0.08353600
C	-6.21562500	-0.66803300	0.18774200

C	-6.66785600	1.73808200	-0.03501100
C	-7.55487400	-0.91204200	0.53244700
C	-4.11463300	-1.10339500	-0.22325300
C	-7.99468000	1.46604200	0.31855400
C	-8.42935600	0.16009500	0.59523600
H	-6.34964800	2.75191700	-0.25663100
H	-7.87423900	-1.92819500	0.74216400
H	-8.70116900	2.28892500	0.37585500
H	-9.46808600	-0.00897200	0.86172100
H	-2.98076800	-2.80937400	-0.14662200
Cu	-2.01869600	0.95645600	0.08970300

Table.S6. XYZ coordinates for calculated optimized geometry of structure of **S-3** at the PBE0/6-31+G(d) level of theory.

O	2.03019200	0.61583400	0.08549100
O	-0.12370600	0.86314000	0.17241600
N	6.77300700	0.59285600	-0.06374300
C	5.61310400	-0.06552600	-0.09053000
C	8.06898600	-0.07243900	-0.26099900
C	6.84950800	2.04019500	0.16704900
C	4.36334200	0.62332800	0.01440700
C	3.14719800	-1.50990900	-0.13517300
C	3.20220100	-0.09093700	-0.00967700

C	5.57585300	-1.50384000	-0.22196800
C	4.40059400	-2.18618500	-0.23991400
C	8.66548100	-0.60303800	1.03576600
C	6.75401200	2.85078500	-1.11906200
C	1.91171100	-2.12109900	-0.14709000
C	0.71847600	-1.37264000	-0.04606400
C	0.82682900	0.04282100	0.07030700
C	-0.54542700	-2.03023400	-0.05704500
H	7.96022800	-0.86048700	-1.01046200
H	8.73216200	0.67505500	-0.70624600
H	6.07946900	2.32951100	0.88738700
H	7.80811500	2.22443000	0.66127700
H	4.30756100	1.70188100	0.08762400
H	6.50111800	-2.06207900	-0.28483900
H	4.40532700	-3.26925800	-0.32899100
H	8.80958000	0.19980100	1.76598600
H	8.03034800	-1.36802900	1.49397100
H	9.64416900	-1.04920900	0.83335100
H	7.55337200	2.58338800	-1.81742900
H	5.79636300	2.69866000	-1.62765200
H	6.85450800	3.91634800	-0.89008200
H	1.85180300	-3.20412900	-0.23843400

H	-0.53969900	-3.12058800	-0.13347500
S	-5.58556500	-1.94345300	-0.08471700
N	-3.85040700	-0.01403200	-0.00828000
N	-2.86445900	-2.10485700	0.01487000
N	-1.68709800	-1.41400700	0.01996600
C	-6.17151800	-0.28613200	-0.09705000
C	-5.09722500	0.61835900	-0.06310500
C	-7.49328500	0.14159700	-0.16243000
C	-5.34243300	1.98995900	-0.12781500
C	-3.97869400	-1.32846400	-0.03581100
C	-7.72517600	1.51215700	-0.19760200
C	-6.66171600	2.42255400	-0.18950400
H	-8.31700900	-0.56522300	-0.18989600
H	-4.52401900	2.70161800	-0.17420200
H	-8.74646800	1.87704000	-0.24686000
H	-6.87007100	3.48671200	-0.24596000
H	-2.87889100	-3.08670600	-0.23912600
Cu	-1.99314700	0.50494700	0.18790900
O	-2.19047300	2.42564000	0.71266200
H	-1.33040400	2.85912800	0.84698300
H	-2.77359200	2.69942400	1.43881000

Table.S7. XYZ coordinates for calculated optimized geometry of structure of **S-4** at the PBE0/6-

31+G(d) level of theory.

O	2.12289500	0.57873900	0.09701500
O	-0.03199800	0.82878200	0.14144700
N	6.86952000	0.55718100	0.05973500
C	5.70846400	-0.10041700	-0.00901800
C	8.16837600	-0.10487000	-0.12151500
C	6.93954900	1.99829800	0.32282300
C	4.45806000	0.58601400	0.08250600
C	3.24393700	-1.53980300	-0.14774800
C	3.29577000	-0.12664500	0.01329800
C	5.67347000	-1.53397400	-0.17437700
C	4.49738000	-2.21441500	-0.23787300
C	8.73459600	-0.66525600	1.17623900
C	6.86751500	2.83667200	-0.94702900
C	2.00529700	-2.14858700	-0.20144600
C	0.81264100	-1.40566600	-0.11038500
C	0.91486000	0.00958300	0.04143100
C	-0.44998100	-2.07293500	-0.15262600
H	8.07724000	-0.87616200	-0.89064800
H	8.84319300	0.65069300	-0.53476900
H	6.15504300	2.27190500	1.03388300
H	7.88774300	2.17420200	0.83982400

H	4.40063000	1.66239200	0.18172400
H	6.59924400	-2.09242700	-0.22747700
H	4.50400800	-3.29511200	-0.35262100
H	8.86032200	0.12106700	1.92762300
H	8.08847400	-1.43983900	1.60176100
H	9.71802000	-1.10730400	0.98797600
H	7.68158700	2.58576500	-1.63450300
H	5.92091600	2.69132800	-1.47780900
H	6.95947800	3.89773800	-0.69449200
H	1.94797000	-3.22945900	-0.31821700
H	-0.43222600	-3.16247900	-0.24266900
S	-5.48890100	-2.05403800	0.00063800
N	-3.78107900	-0.10001700	0.05744800
N	-2.76224700	-2.16235800	-0.11787900
N	-1.59219800	-1.46419000	-0.08551900
C	-6.09859100	-0.40750300	0.10125500
C	-5.03507500	0.51073400	0.10958500
C	-7.42559800	0.00508200	0.15590000
C	-5.29617900	1.88075500	0.15550900
C	-3.88766800	-1.41200900	-0.01424700
C	-7.67340800	1.37277500	0.21195900
C	-6.62174500	2.29701000	0.20786900

H	-8.24250100	-0.71016300	0.15139000
H	-4.48330600	2.60154600	0.13769300
H	-8.69954600	1.72480200	0.25428000
H	-6.84524500	3.35913800	0.24255200
H	-2.76675300	-3.17508500	-0.07078100
Cu	-1.92115300	0.48169100	0.06305000
O	-2.13711600	2.21553800	1.18615900
H	-1.28227400	2.59490900	1.44646300
H	-2.70525700	2.23465300	1.97188400
O	-2.02186100	1.51922100	-1.89536300
H	-2.83728300	1.66979300	-2.39567200
H	-1.39697600	2.20205600	-2.17927200

Table.S8. The free energies of reactions (kcal.mol-1) to form the **S-1, S-2, S-3** and **S-4** configurations at the PBE0/6-31+G(d) level of theory.

**Reactions**	ΔG_{aq}^{0}**(kcal.mol-1)**
$\left[Cu(H_2O)_5\right]_{(aq)}^{2+} + L_{(aq)} \rightarrow [CuL]_{(aq)}^{2+}(S\text{–}1) + 5H_2O_{(aq)}$	-39.6
$\left[Cu(H_2O)_5\right]_{(aq)}^{2+} + L_{(aq)} \rightarrow [CuL]_{(aq)}^{2+}(S\text{–}2) + 5H_2O_{(aq)}$	-7.2
$\left[Cu(H_2O)_5\right]_{(aq)}^{2+} + L_{(aq)} \rightarrow \left[CuL(H_2O)\right]_{(aq)}^{2+}(S\text{–}3) + 4H_2O_{(aq)}$	-34.8
$\left[Cu(H_2O)_5\right]_{(aq)}^{2+} + L_{(aq)} \rightarrow \left[CuL(H_2O)_2\right]_{(aq)}^{2+}(S\text{–}4) + 3H_2O_{(aq)}$	-25.8

$[Cu(H_2O)_5]^{2+}$ is the most stable form of complex between Cu^{2+} ion and water [*]

[*] FRANK, Patrick, et al. The solution structure of $[Cu(aq)]^{2+}$ and its implications for rack-induced bonding in blue copper protein active sites. Inorganic chemistry, 2005, 44.6: 1922-1933.

Table.S9. The structural parameters of **S-1**, **S-2**, **S-3**, and **S-4** at the PBE0/6-31+G(d) level of theory (bond lengths in angstrom, angles in degrees)

Bond lengths	**S-1**	**S-2**	**S-3**	**S-4**	**L**
O2–Cu	1.85	1.95	1.90	1.92	
N34–Cu	1.90		1.94	1.95	
N36–Cu	1.95	2.12	1.95	1.98	
S33–Cu		2.23			
Cu–O(H_2O)			2.00	2.08	
Cu–O(H_2O)				2.22	
Bond angles	**S-1**	**S-2**	**S-3**	**S-4**	
O2–Cu–N34	174.93		172.12	172.71	
O2–Cu-N36	94.89	89.93	91.74	90.99	
N34–Cu–N36	84.14		83.00	82.24	
S33–Cu–N36	84.71				
S33–Cu–O2	164.62				
O(H_2O)–Cu–O(H_2O)				94.72	
Dihedral angles					
Cu–O2–N36–N34	3.65		4.39	2.01	
Cu–O2–N36–S33	10.87				
O(H_2O) –O2–N36–N34			12.18	19.15	
O(H_2O) –O2–N36–N34				-47.66	
C15 –C17–N36–N35	-175.6	-176.5	-179.5	-179.3	179.9
C17–N36–N35–C41	174.7	-179.7	-173.6	178.8	179.9
N36–N35–C41–N34	-3.6	-160.4	-3.3	2.5	179.9
N36–N35–C41–S33	173.3	17.7	178.7	-178.8	-0.5
N35–C41–N34–C38	176.0	170.0	-179.8	177.8	179.9
N35–C41–S33–C37	-176.7	-171.9	179.0	-178.3	-180.0

Fig.S5. The optimized geometries of Histamine at the PBE0/6-31+G(d) level of theory

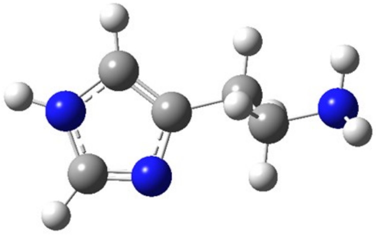

Fig.S6. The optimized geometries of $[Cu(\mathbf{L}_{ref})(H_2O)_2]^{2+}$ ($\mathbf{L}_{ref}$: histamine) at the PBE0/6-31+G(d) level of theory.

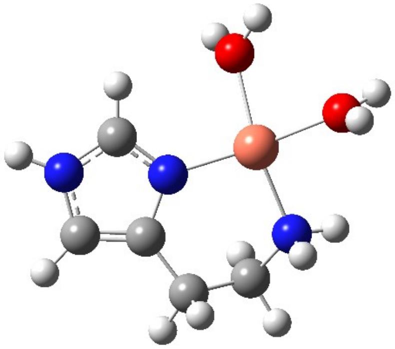

Table.S10. XYZ coordinates for calculated optimized geometry of structure of Histamine at the PBE0/6-31+G(d) level of theory.

C	2.20218400	-0.91019300	-0.03018200
C	1.35644500	1.11934200	-0.09919000
C	0.39153800	0.20574000	0.24997000
N	0.93802800	-1.05747500	0.28594700
H	3.41441200	0.75516700	-0.52859300
H	1.32750400	2.19163300	-0.23026000
N	2.50945000	0.39053500	-0.27328000
C	-1.05173800	0.43731200	0.55418900

H	-1.28528700	0.08549800	1.56690200
H	-1.25731400	1.51658800	0.53616300
C	-1.98158900	-0.28164700	-0.42124000
H	-1.74898100	0.04792100	-1.44939700
H	-1.76828900	-1.35487500	-0.37447000
N	-3.37302100	-0.07658000	-0.03548000
H	-3.64820500	0.89107700	-0.18347300
H	-3.99178000	-0.64828500	-0.60272800
H	2.93569700	-1.70341100	-0.09574500

Table.S11. XYZ coordinates for calculated optimized geometry of structure of Cu(**L**$_{ref}$)(H_2O)$_2$]$^{2+}$ at the PBE0/6-31+G(d) level of theory.

C	1.38185500	-1.70782300	0.12336100
C	3.02725000	-0.24076400	0.14985200
C	1.84275000	0.43967200	0.09237300
N	0.81994600	-0.49716600	0.07110600
H	3.37132900	-2.33947300	0.21917100
H	4.04917400	0.10955200	0.18414000
N	2.70742500	-1.57368500	0.17317700
C	1.60889400	1.91535700	0.11385500
H	1.54859000	2.27198900	1.15313500
H	2.47790000	2.42093000	-0.31976000
C	0.37375200	2.35856700	-0.64924800

H	0.32349300	3.45278800	-0.66992900
H	0.38885000	2.00312800	-1.68408200
N	-0.86636200	1.83889700	-0.00344700
H	-0.91497800	2.19768400	0.95456500
H	-1.68332500	2.23511000	-0.47520700
H	0.86315400	-2.65519000	0.13473900
Cu	-1.06844600	-0.13462700	0.01554800
O	-1.55450300	-1.98840800	-0.65871200
H	-1.31097000	-2.34108200	-1.53061000
H	-2.34572400	-2.46730600	-0.36096800
O	-3.02958400	-0.02684700	0.62078000
H	-3.33019000	0.13801500	1.53059400
H	-3.81374600	0.03372200	0.04977600

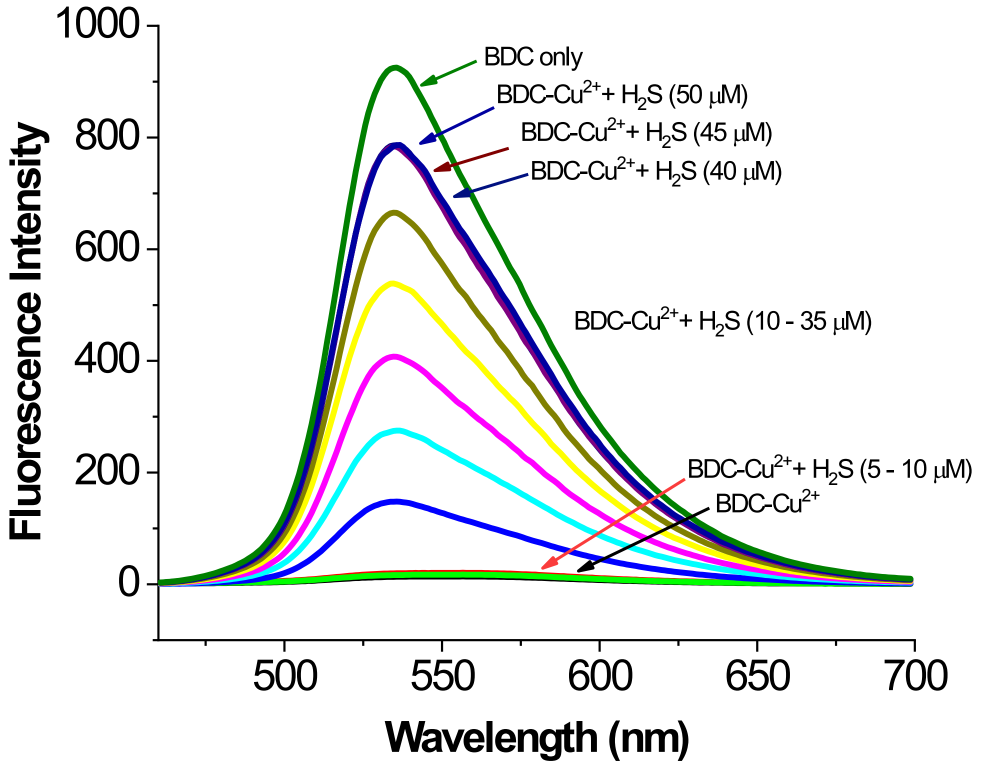

Fig.S7. Fluorescence spectra of BDC (5 μM); BDC (5 μM)+Cu^{2+} (5 μM); BDC (5 μM)+ Cu^{2+} (5 μM) + H_2S (5, 10, 15, 20, 25, 30, 35, 40, 45, 50 μM).

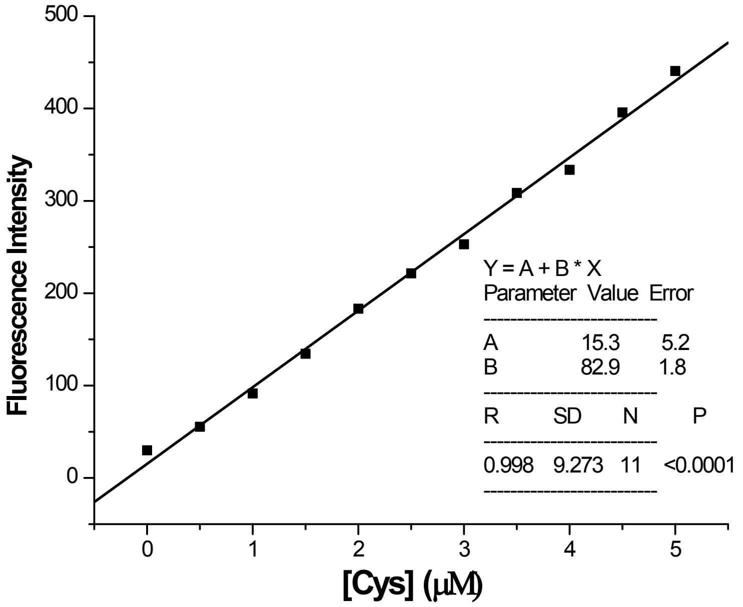

Fig.S8. Variation of fluorescence intensity of **BDC**-Cu^{2+} vs the concentration of Cys (for calculation of the detection and quantitation limits)
